# Expression and potential role of FOSB in glioma

**DOI:** 10.3389/fnmol.2022.972615

**Published:** 2022-10-12

**Authors:** Min Qi, Le-an Sun, Lan-rong Zheng, Jia Zhang, Yan-ling Han, Feng Wu, Jian Zhao, Wen-hao Niu, Mao-xing Fei, Xiao-chun Jiang, Meng-liang Zhou

**Affiliations:** ^1^The Translational Research Institute for Neurological Disorders of Wannan Medical College, Department of Neurosurgery, The First Affiliated Hospital of Wannan Medical College (Yijishan Hospital of Wannan Medical College), Wuhu, China; ^2^Department of Neurosurgery, Jinling Hospital, School of Medicine, Nanjing University, Nanjing, China; ^3^Department of Pathology, Wannan Medical College, Wuhu, China; ^4^Department of Anatomy, Wannan Medical College, Wuhu, China

**Keywords:** glioma, proliferation, migration, apoptosis, FOSB

## Abstract

**Background:**

FOSB is reported to be an oncogene in a variety of tumors. However, the expression and role of FOSB in glioma remain obscure. In this study, we aimed to explore the expression of FOSB in glioma and its biological role in glioblastoma multiforme (GBM).

**Methods:**

Western blot, immunohistochemical staining, and quantitative real-time polymerase chain reaction (RT-qPCR) were used to detect the expression of FOSB in clinical samples. FOSB was knocked down in cells to determine the effects of FOSB on the phenotypic changes of tumors by plate cloning, CCK-8 assay, and Transwell assay. Finally, subcutaneous tumorigenesis in nude mice was used to observe the tumorigenesis of glioma cell lines after the knockdown of the FOSB gene.

**Results:**

FOSB expression was higher in glioma compared with normal brain tissue. After the downregulation of FOSB, the expression of cleaved caspase-3 increased. Plate cloning and CCK-8 experiments showed that the proliferation of glioma cell lines decreased. The Transwell assay demonstrated that the glioblastoma cell lines had lower migration ability after the knockdown of FOSB. Finally, the tumor volume of *U87* glioma cells in group sh-FOSB was smaller than that in the control group. The TUNEL staining *in vitro* showed that the apoptosis of sh-FOSB glioma cells increased.

**Conclusion:**

FOSB was highly expressed in glioma tissues. The viability of glioma cells decreased, and the ability of glioma cells to proliferate and migrate was reduced when FOSB was downregulated. Hence, FOSB may promote the development and migration of gliomas.

## Introduction

Glioma is the most common subtype of primary brain tumors in adults. Malignant glioma, especially GBM, is aggressive, highly invasive, and neurologically destructive. The current standard for treating glioma patients includes surgery combined with radiation and chemotherapy. Temozolomide (TMZ) is a first-line treatment for glioblastoma. However, the prognosis for patients with GBM is dismal, with a median survival of only 15 months and a 5-year survival rate of <10%. Therefore, identifying critical molecules that regulate the development, invasion, and recurrence of GBM *via* targeted therapy is necessary (Bandey et al., 2015; Liu et al., 2015; Chen et al., 2017; Court et al., 2019; Huang et al., 2019; Wang et al., 2021; Tsai et al., 2022). FOSB is a member of the multi-gene Fos family, which has an important role in regulating cell growth and proliferation. Its homologs include c-Fos, Fra-1, and Fra-2. The members of the Fos family can form activator protein-1 (AP-1) with Jun protein dimer (Milde-Langosch, 2005; Papoudou-Bai et al., 2017; Prucca et al., 2020). AP-1 transcriptional factors were either absent or weakly expressed in normal human astrocytes and normal brain specimens. However, AP-1 was overexpressed in human gliomas and GBM cell lines. Overexpression of AP-1 could promote the development of tumors (Ahn et al., 2016; Bhardwaj et al., 2018). FOS was found to be involved in the progression of malignant glioma (Tao et al., 2013). C-Fos overexpression is inversely correlated with the survival time of patients with gliomas, which could affect the cell cycle, apoptosis, and radiosensitivity of *T98G* and *U251* GBM cell lines (Liu et al., 2016). Additionally, Fra1 upregulation can improve the invasiveness and drug resistance of glioma (Meise et al., 2012; Zhang et al., 2017). Fra-2 could promote cell proliferation, migration, and invasion. The expression of Fra-2 was closely related to the prognosis of patients with glioma (Luo et al., 2018). The FOSB gene has been reported to be a proto-oncogene. A previous study found that FOSB silencing inhibited proliferation, migration, and invasion of pancreatic cancer cells (Liu et al., 2018). FOSB assumes an oncogenic role in the pathogenesis of endothelial tumors, bladder cancer, and other tumors (Hung et al., 2017; Eissa et al., 2019). It has been reported that FOSB upregulation was associated with poor outcomes in the TCGA-GBM cohorts (Rowther et al., 2016). However, there are no studies on the expression and mechanisms of FOSB in glioma.

The present study determined the expression of FOSB in glioma tissues and cell lines. The underlying mechanisms were also investigated, which may provide potential targets for GBM treatment in the future.

## Materials

### Reagents and antibodies

Antibodies against FOSB, GAPDH, and cleaved caspase-3 were purchased from Cell Signaling Technology (Beverly, MA, USA). The catalog numbers were #2251, #5174, and #9661. Antibodies against caspase-3 (Cat no. 19677-1-AP), Bcl-2 (Cat no. 26593-1-AP), and Bax (Cat no. 50599-1-lg) were purchased from Proteintech Co., Ltd. (Wuhan, China). Phosphatase inhibitors were purchased from Abcam (Cambridge, UK). The catalog numbers were #ab184938. FOSB (Cat NO. bsm-52071R) was purchased from Beijing Bioss Biological Co., Ltd. for Immunohistochemistry. DAPI (Cat NO. KGA215) was purchased from Nanjing Kaiji Biological Co., Ltd.

### Clinical samples and cell lines

From October 2017 to June 2021, 72 glioma tissue specimens were collected from Yijishan Hospital of Wannan Medical College, including 16 cases of grade II, 35 cases of grade IV, and five cases of normal brain tissue specimens. None of the patients with glioma received any antineoplastic treatment before surgery. Postoperative pathology was graded according to WHO scoring criteria. The pathological grading was based on the diagnostic results of the Pathology Department of Yijishan Hospital. After tissue excision, all specimens were collected directly. Half of each tissue specimen was preserved in 4% polyformaldehyde for future paraffin embedding, and the other half was rapidly frozen in liquid nitrogen. The patients did not receive anticancer treatment, and they gave signed informed consent before surgery.

Human GBM cell lines U87, U251, A172, U118, and T98G and a human astrocyte (HA) line were obtained from the American Typical Culture Preservation Center (ATCC) in February 2019. These cell lines have been tested and authenticated. RNA was extracted from the genomic extract kit of Axygen, amplified by the 20-STR amplification protocol, and STR and gender Amelogenin were detected on the ABI 3730XL genetic analyzer. Cells were cultured in DMEM (containing 10% fetal bovine serum, 4,500 mg/L d-glucose and l-glutamine, 1% penicillin and streptomycin) at 37°C and 5% CO_2_. The fetal bovine serum we used was from Sigma-Aldrich, CAS-No 1943609-65-1. The last tested time was December 10, 2021.

## Methods

### Immunohistochemical and immunofluorescence analysis

Immunohistochemistry on formalin-fixed paraffin-embedded sections was performed to determine the immunoreactivity of FOSB. Sections were deparaffinized and rehydrated in graded concentrations of ethanol in distilled water. Endogenous peroxidase activity was blocked with 3% H_2_O_2_ for 5 min, followed by a brief rinse in distilled water and a 15-min wash in PBS. Sections were placed in 10 mmol/L citrate buffer (pH 6.0), heated in a microwave oven at 95°C for 30 min, then cooled down to room temperature for 20 min, and rinsed in PBS. Non-specific protein binding was blocked by a 40 min incubation in 5% horse serum. Sections were incubated with primary antibodies (anti-FOSB diluted 1:100; Beijing Bioss Biological Co., Ltd.) for 16–18 h at 4°C, followed by a 15-min wash in PBS. Sections were incubated with HRP-conjugated goat anti-rabbit IgG (1:500 dilution) for 60 min at room temperature. Diaminobenzidine (DAB) was used as a chromogen, and counterstaining was performed with hematoxylin.

Immunofluorescence was used to detect the immunoreactivity of FOSB in formalin-fixed paraffin-embedded sections. After dewaxing, antigen repair, and cell lysis, the slides were incubated with FOSB polyclonal antibody (Abcam, Cambridge, USA) for 16–18 h at 4°C before washing in phosphate buffer saline (PBS) with 1.6% hydrogen peroxide. After PBS was removed, DAPI dye solution was added (Nanjing Kaiji Biological Co., Ltd.) and incubated at room temperature for 10 min. After washing with PBS, the slides were incubated with goat anti-rabbit IgG (diluted 1:500; Santa Cruz Biotechnology, Santa Cruz, CA, USA) and horseradish peroxidase (HRP) for 60 min at room temperature. The slices were washed three times with PBS (pH 7.4) for 5 min each. The slices were then slightly dried and sealed with anti-fluorescence quenching. The sections were observed under a fluorescence microscope, and the images were collected.

### Hematoxylin-eosin staining

Paraffin sections were dewaxed in xylene and then immersed in absolute ethanol, 95% ethanol, 85% ethanol, 75% ethanol, and distilled water to complete the hydration process. The slices were then stained with hematoxylin staining solution, rinsed out the excess hematoxylin (Roche, H04445) stain, immersed in PBS and 95% ethanol, and finally stained with eosin (Roche, G30859) stain. Seventy percent ethanol was dehydrated, transparent, and sealed after washing.

### RT-qPCR analysis

In keeping with the manufacturer's instructions, total RNA was isolated from tissue samples or cultured cells using Trizol reagent (Invitrogen), and reverse DNA transcription was undertaken immediately. Gene-specific primers were follows: FOSB (forward: *ACCCTCTGCCGAGTCTCAATAT*; reverse: *GCCACTGCTGTAGCCACT CAT*); ACTB (forward: *CACCCAGCACAATGAAGATCAAGAT*; reverse: *CCAGTT TTTAAATCCTGA GTCAAGC*). Data analysis was performed using the ΔΔCt method (threshold cycle normalized by ACTB compared with the control), and the fold-change was calculated as 2^−Δ*ΔCt*^.

### Cell transfection and the establishment of stable cell lines

The *U87* and *U251* cell lines were cultured in DMEM (containing 10% FBS, 1% penicillin, and streptomycin) at 37°C and 5%, respectively. A downregulated FOSB cell model was established by lentiviral transfection. A human FOSB knockdown lentivirus vector was designed and produced by China Shanghai Hambio Co., Ltd. Lentiviruses were used to knock down FOSB or as negative controls. The interference sequence shRNA was: *CCGCCAGGCGGACAGATCAGTCT CGAACTGATCTGTCTCCGCCTGGTTT*. Cells were transfected according to the manufacturer's instructions. The catalog number of lentivirus was HH20190619RFF-LV01-3. We labeled both control and interfering viruses with ZsGreen-PURO. After successful transfection, the virus could survive in the medium containing puromycin, and these transfected cells carried green fluorescent protein (GFP) and displayed green fluorescence. After 48 h transfection, a puromycin-containing medium was added for screening. Cell passaging was conducted for 2–3 generations to establish stably transfected cell lines, which were frozen at −80°C for future laboratory use.

### Western blot

Frozen brain tissue was mechanically lysed in 20 mM Tris pH 7.6 containing 0.2% SDS, 1% Triton X-100, 1% deoxycholate, one mM phenylmethylsulphonyl fluoride (PMSF), and 0.11 IU/ml aprotinin (all purchased from Sigma-Aldrich, Inc., St. Luis, MO, USA). Lysates were centrifuged at 12,000 × g for 20 min at 4°C. The protein concentration was estimated by the Bradford method using the Nanjing Jiancheng (NJJC) protein assay kit (Nanjing Jiancheng Bioengineering Institute, Nanjing, China). The samples (60 μg per lane) were separated by 8% SDS-PAGE and electro-transferred onto polyvinylidene-difluoride (PVDF) membranes (Bio-Rad Lab, Hercules, CA, USA) and incubated with primary antibodies against FOSB (1:1,000), Caspase-3 (1:1,000), Cleaved Caspase-3 (1:1,000), Bcl-2 (1:1,000), Bax (1:1,000) and with GAPDH (1:5,000) as a loading control. After the membranes were washed six times for 10 min each in PBST, they were incubated with the appropriate HRP-conjugated secondary antibody (1:400) for 2 h. The blotted protein bands were wetted with chemiluminescence HRP substrate (Millipore, Burlington, MA, USA). Moreover, the Amersham ImageQuant 800 (Cytiva Sweden AB, Uppsala, Sweden) was used to expose the strip immediately. All experiments were repeated at least three times.

### Plate cloning experiments

Cells in the logarithmic growth phase were digested with 0.25% trypsin and separated into single cells. Cells were suspended in a DMEM medium containing 10% FBS and were diluted and counted. The cell suspension volume was calculated by adding 3,000 cells to each culture dish with a 10 ml total culture medium. After 3–4 weeks of culture, the experiment was completed when visible colonies were observed.

### Transwell assay

Twenty-four well plate cell chambers (Corning) with an aperture of 8 μl were selected for the Transwell experiment. Cells suspended in 100 μl of serum-free medium (10^5^ cells/well) were added to the upper chamber, and 500 μl of medium containing 10% fetal bovine serum (FBS) was added to the lower chamber. After 24 h of culture, the cells that did not pass through the hole were gently wiped with a cotton swab, fixed with 4% paraformaldehyde, and stained with crystal violet. After drying, microscope observation and photographing were carried out. After drying, the blade chamber cut was observed and photographed with a microscope (Leica, RM2265).

### MTT assay

The cells were cultured in 96 healthy plates, and 10 μl MTT (5 mg/ml) was added to each well on the second day after adhesion and cultured for 3–4 h. Then, it carefully absorbed the original medium. One hundred fiftymicro-liter of DMSO (Beyotime Biotechnology Co., Ltd.) was added to each well and incubated at 37°C for 10 min. Then, the absorbance (A) value of each hole was detected by an enzyme labeling instrument (The OD value was 490 nm). Cell viability of control = (a value of experimental group – a value of zeroing hole)/(a value of control hole – a value of zeroing hole) ^*^ 100%. Set zero adjustment and control holes (wild-type *U87* or wild-type *U251* cell line).

### Cell proliferation assay

A cell counting kit 8 (CCK-8 kit) was purchased from the Japan Tongren Institute of Chemistry for cell survival and proliferation detection. Glioblastoma cells in logarithmic proliferation were cultured in 96-well cell culture plates. Cells were seeded at a density of 2,000 cells/well in a 100 μl complete culture medium. After culturing for 72 h, the culture medium was discarded, and 100 μl of complete culture medium containing 10% CCK-8 solution was added and incubated at 37°C for 2 h. Absorbance was measured at 450 nm in a plate reader (BioRad, Berkeley, CA, USA), and the relative cell proliferation rate was calculated.

### Subcutaneous implantation model of GBM in nude mice

The animal experiments in this study were approved by the Institutional Animal Care and Use Committee (IACUC) of Yijishan Hospital (Wuhu, China) and followed the Guidelines for the Care and Use of Laboratory Animals from the Chinese Council on Animal Research. Specifically, 4-week-old male BALB/C nude mice were purchased from GemPharmatech Co., Ltd. (Nanjing, China) and kept in an SPF-level Laboratory Animal Room. Mice received SPF mouse food and were allowed to drink sterile water freely. For our experiment, sh-FOSB or sh-Ctrl GBM cells were grown separately in the logarithmic growth phase, digested with trypsin, and harvested in PBS. These cell suspensions were then inoculated subcutaneously into nude mice (10^6^ cells per nude mouse). This way, the steps to establish a subcutaneous graft tumor model of nude mice were completed. Then, the tumor formation and growth of tumor cells were monitored for 28 days. Nude mice with subcutaneous tumors were measured every 2–3 days. The calculation formula is v = ABB/2 (“A” is the long diameter and “B” is the short diameter). On the 28th day of subcutaneous tumor implantation, nude mice bearing a subcutaneous tumor model were photographed by the Clinx IVScope 8,500 small animal *in-vivo* image system. At the end of the experiment, the mice were anesthetized by inhaling isoflurane (induced at 4% and maintained at 1.5%). The tumors in the experimental and control groups were stripped and photographed. The stripped subcutaneous tumor was fixed with 10% neutral formalin. Conventional dewaxed sections were embedded in paraffin, and 4 μm thick sections were prepared by a paraffin slicer (Leica, RM2265) for further experiment.

### TUNEL assay

The sections were subjected to TUNEL labeling and DAPI staining of the cell nuclei according to the kit instructions (Nanjing Kaiji Biological Co., Ltd.). The stained sections were photographed under the fluorescence microscope (Zeiss, Axio Scope A1).

### Statistical analysis

Comparisons between different groups were performed by analysis of variance (ANOVA) followed by Tukey's multiple comparisons test if a significant difference had been determined by ANOVA. A probability value of *P* < 0.05 was considered statistically significant.

## Results

### Expression of FOSB in human glioma tissue and glioma cells

**Figure 1:**
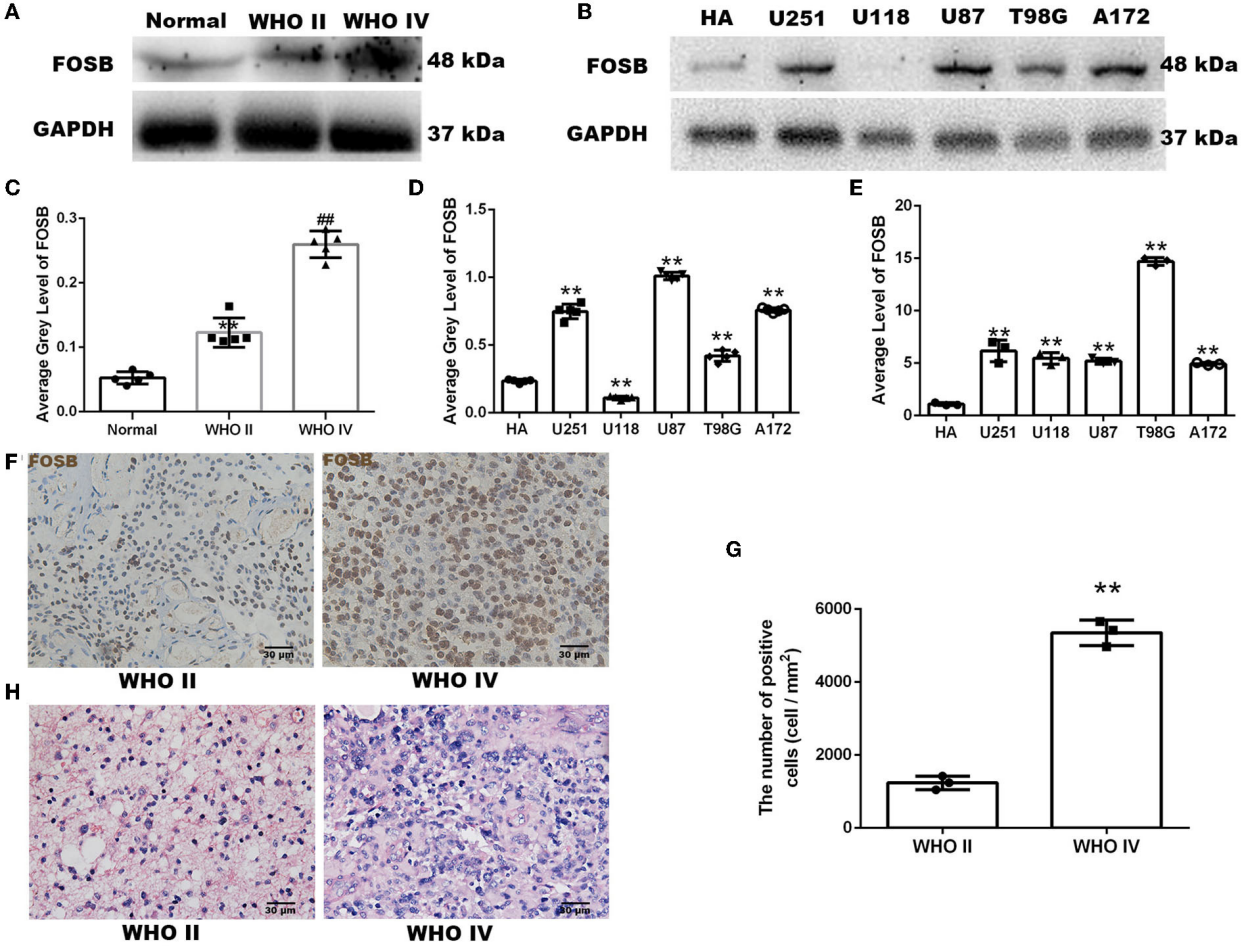
Expression of FOSB in human glioma samples and glioma cells. **(A)** FOSB expression in normal brain samples, low-grade glioma, and high-grade glioma samples was determined by Western blot (*n* = 5 each). **(B)** FOSB expression in normal human astrocytes and five glioma cell lines (*U251, U118, U87, T98G*, and *A172*) was compared *in vitro* (*n* = 3). **(C)** The quantitative analysis of **(A)** showed high FOSB expression in high-grade gliomas (*n* = 5, **P < 0.01 vs. normal brain tissues; ^##^*P* < 0.01 vs. WHO II glioma tissues). **(D)** The quantitative analysis of **(B)** showed higher expression of FOSB in glioma cell lines (*n* = 3, **P < 0.01). **(E)** FOSB expression in cell lines was detected by quantitative reverse transcriptase polymerase chain reaction (RT-qPCR). FOSB expression in glioma cell lines (*U251/U118/U87/T98G/A172*) was also higher than that in human astrocytes (HA; *n* = 3, **P < 0.01). **(F)** Immunohistochemistry was used to detect expression in low-grade and high-grade glioma samples (*n* = 3). **(G)** The positive cell count of **(F)** showed high FOSB expression in high-grade gliomas (*n* = 3, *P < 0.01 vs. low-grade glioma). **(H)** Hematoxylin-eosin staining of low-grade and high-grade glioma (*n* = 5).

A western blot was used to quantitatively analyze the expression of FOSB. FOSB expression in glioma was higher than that in normal brain tissue. Furthermore, the expression of FOSB in high-grade glioma was higher than that in low-grade glioma (^**^P < 0.01 vs. normal brain tissue, ^##^*P* < 0.01 vs. low-grade glioma; Figures 1A,C). Five glioma cell lines were compared with a human astrocyte cell line (HA). Western blot results showed that FOSB expression in *U251, U87, T98G*, and *A172* glioma cell lines was higher than in HA, especially in *U251* and *U87* (Figures 1B,D). Quantitative reverse transcriptase polymerase chain reaction (RT-qPCR) results showed that the expression of FOSB in each of the five glioma cell lines was higher than that in the human astrocyte cell line at the mRNA level (Figure 1E). The discrepancy between protein and gene expression results was observed in the U118 glioma cell line and seems to be due to the time lag effect in protein modification, gene translation, and translation in this particular cell line (Song et al., 2022). Immunohistochemistry and immunofluorescence were used to detect the expression of FOSB in glioma tissues. In immunohistochemical studies, cell staining intensity varied according to grade. High-grade glioma expressed high levels of FOSB (Figures 1F,G). Hematoxylin-eosin (HE) staining showed that the atypia of high-grade glioma was greater than that of low-grade glioma (Figure 1H).

### FOSB could regulate apoptosis of GBM cell lines

**Figure 2:**
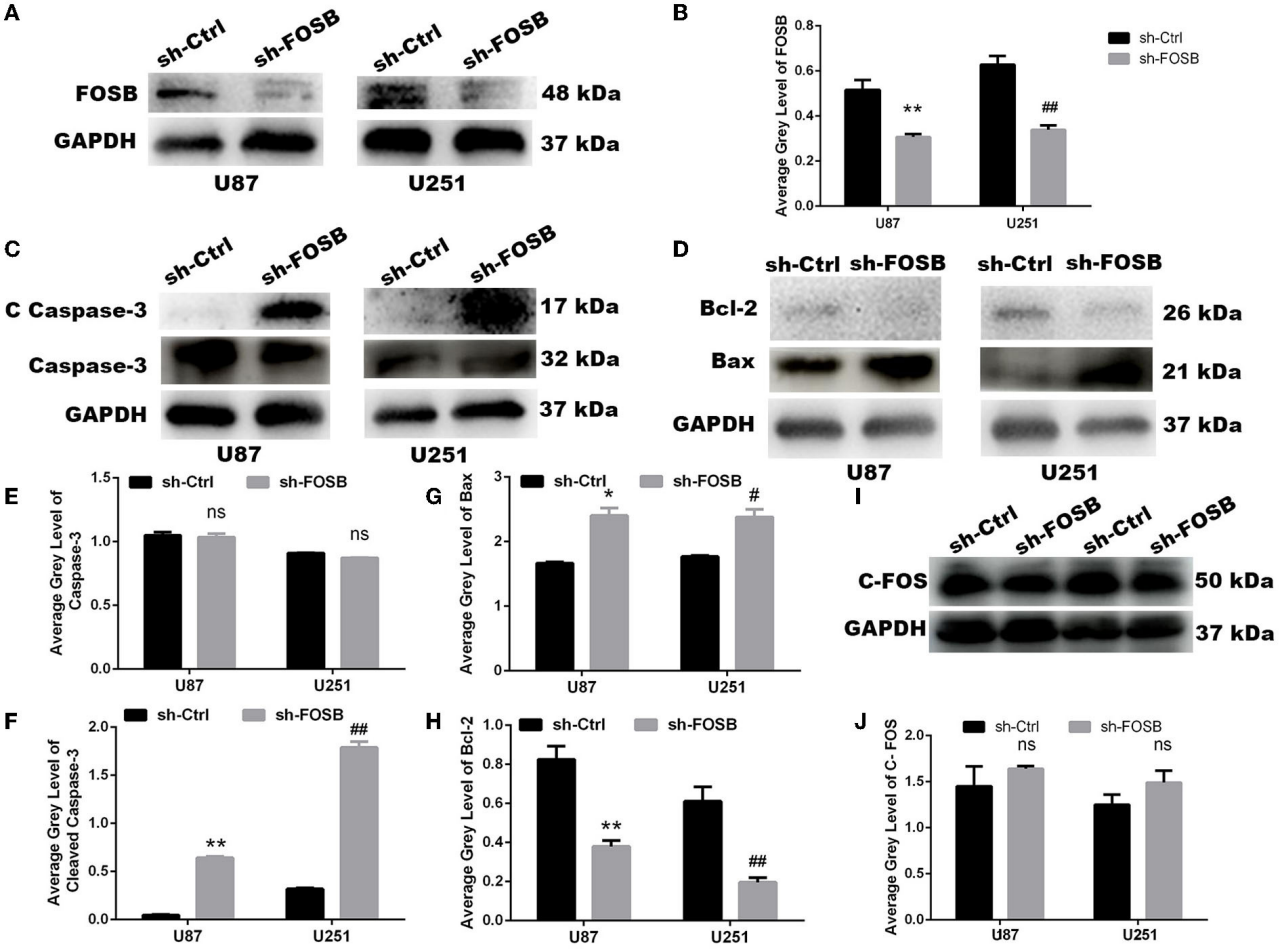
FOSB regulates apoptosis of GBM cell lines. **(A,B)** FOSB expression in *U87* and *U251* cell lines was decreased after knockdown of FOSB (*n* = 3, **P < 0.01 vs. sh-Ctrl of *U87* GBM cell lines, ^##^*P* < 0.01 vs. sh-Ctrl of *U251* cell lines). **(C,E,F)** Expression of Caspase-3 and Cleaved Caspase-3 after knocking down FOSB in *U87* and *U251* cell lines. **(E)** There was no significant difference in the expression of Caspase-3 between sh-FOSB and sh-Ctrl. **(F)** And the expression of Cleaved Caspase-3 was increased (*n* = 3, **P < 0.01 vs. sh-Ctrl of *U87* cell lines, ^##^*P* < 0.01 vs. sh-Ctrl of *U251* cell lines). **(D,G,H)** Expression of Bax and Bcl-2 after knocking down FOSB in *U87* and *U251* cell lines. **(G)** The expression of Bax was increased (*n* = 3, *P < 0.01 vs. sh-Ctrl of *U87* cell lines, ^#^*P* < 0.01 vs. sh-Ctrl of *U251* cell lines). **(H)** At the same time, the expression of Bcl-2 in the sh- FOSB group was significantly lower than that in the sh-Ctrl group. (*n* = 3, **P < 0.01 vs. sh-Ctrl of *U87* cell lines, ^##^*P* < 0.01 vs. sh-Ctrl of *U251* cell lines). **(I,J)** Expression of C-FOS after knocking down FOSB in U87 and U251 cell lines. **(J)** There was no significant difference in the expression of C-FOS between sh-FOSB and sh-Ctrl.

As mentioned above, FOSB expression in *U87* and *U251* cell lines was higher than in other glioma cell lines, and thus *U87* and *U251* cell lines were selected for lentiviral transfection. After the knockdown of FOSB, stably transfected cell lines were established with *U87* and *U251* cells. Western blot experiments showed that FOSB expression was downregulated in *U87* and *U251* cell lines, which indicated that FOSB had been knocked down successfully and that stable transfection cell lines had been successfully established (^**^P < 0.01; Figures 2A,B). Activation of caspase 3 is central to apoptosis (Shoda et al., 2022). The regulatory steps of mitochondrial apoptosis are mediated by the Bcl-2 family of proteins. The Bcl-2 protein has an antagonistic effect on caspase 3 (Castillo et al., 2022). Bax is one of the most important apoptosis genes (Garciaz et al., 2022). Detection of caspase-3, cleaved caspase-3 (Figures 2C,E,F), Bax, and Bcl-2 (Figures 2D,G,H) demonstrated that apoptosis increased significantly after knockdown of FOSB in *U87* and *U251* cell lines. At the same time, the C-FOS did not change due to the knockdown of FOSB, indicating that no relevant potential compensatory effects occurred (Figures 2I,J).

### Knockdown of FOSB and changes in tumor phenotype

**Figure 3:**
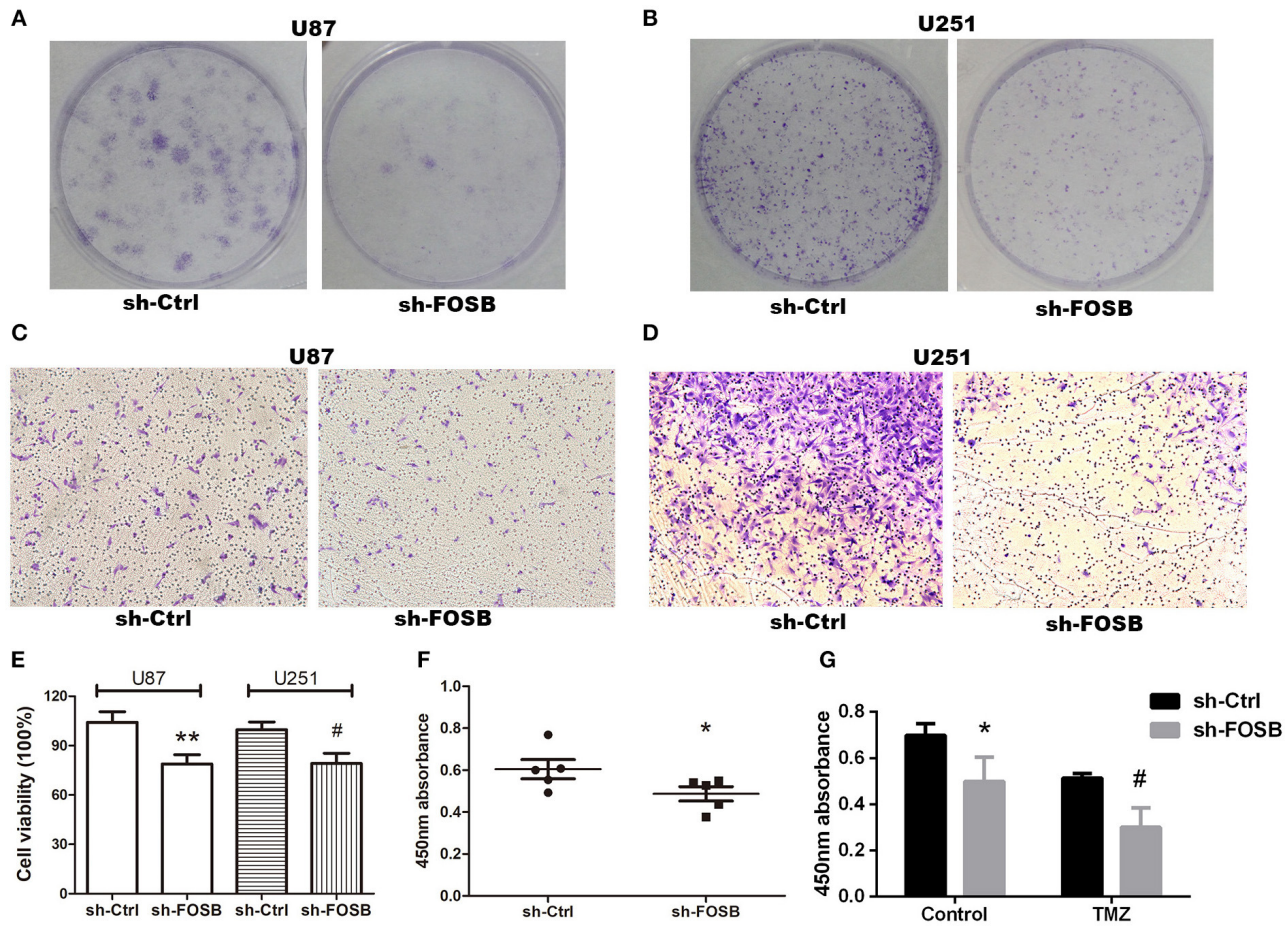
Effects of FOSB on glioma phenotypes. **(A,B)** The proliferative ability of *U87* and *U251* cell lines was detected by plate cloning. **(C,D)** The invasive ability of *U87* and *U251* cell lines after downregulation of FOSB was studied by Transwell assay. **(E)** MTT assay showed that the cell viability of *U87* and *U251* cells after FOSB knockout decreased. (**P < 0.05 vs. sh-Ctrl of *U87* cell lines, ^#^*P* < 0.05 vs. sh-Ctrl of *U251* cell lines). **(F)** CCK-8 assay was used to detect the proliferation rate of *U87* cell lines after the knockdown of FOSB. (*P < 0.05 vs. sh-Ctrl of *U87* cell lines). **(G)** *U87* cell lines with knocked down FOSB were treated with TMZ, and cell proliferation was detected 72 h after treatment to detect the response of stabilized transfected cell lines to antineoplastic drugs. The control reagent was DMSO, which was the solvent of TMZ (^*^P < 0.05 vs. sh-Ctrl of the control group (DMSO) *U87* cells; ^#^*P* < 0.05 vs. sh-Ctrl of TMZ group *U87* cells).

Glioma is a malignant brain tumor with rapid proliferation and high invasion. TMZ is mostly used in clinical treatment to inhibit tumor recurrence. Hence, we attempted to detect whether FOSB had any effects on the proliferation and invasion of glioma cells and whether the resistance of glioma cell lines to TMZ was affected by FOSB knockdown. Plate cloning was used to detect the proliferation of glioma cell lines. Compared with the control, knockdown of FOSB could significantly inhibit the proliferation of glioma cell lines (Figures 3A,B). The invasive ability of glioma cell lines was studied by the Transwell assay. As shown in Figure 3, FOSB knockdown significantly inhibited the invasion of glioma cells compared with the control group (Figures 3C,D). Cell viability was detected by the MTT assay. As shown in Figure 3E, cell viability increased after FOSB knockout compared with the control group (Figure 3E). In addition, the CCK-8 experiment also confirmed that the proliferation of glioma cell lines decreased after the knockdown of FOSB (Figure 3F). The CCK-8 assay was also used to detect changes in drug resistance after knockdown of FOSB. Compared with the control group (DMSO group), TMZ could inhibit the proliferation of tumors. Interestingly, after the knockdown of FOSB, the proliferation of gliomas was further inhibited. At the same time, resistance to TMZ decreased (Figure 3G).

### Knockdown of FOSB could inhibit the growth of subcutaneous tumor-bearing nude mice

**Figure 4:**
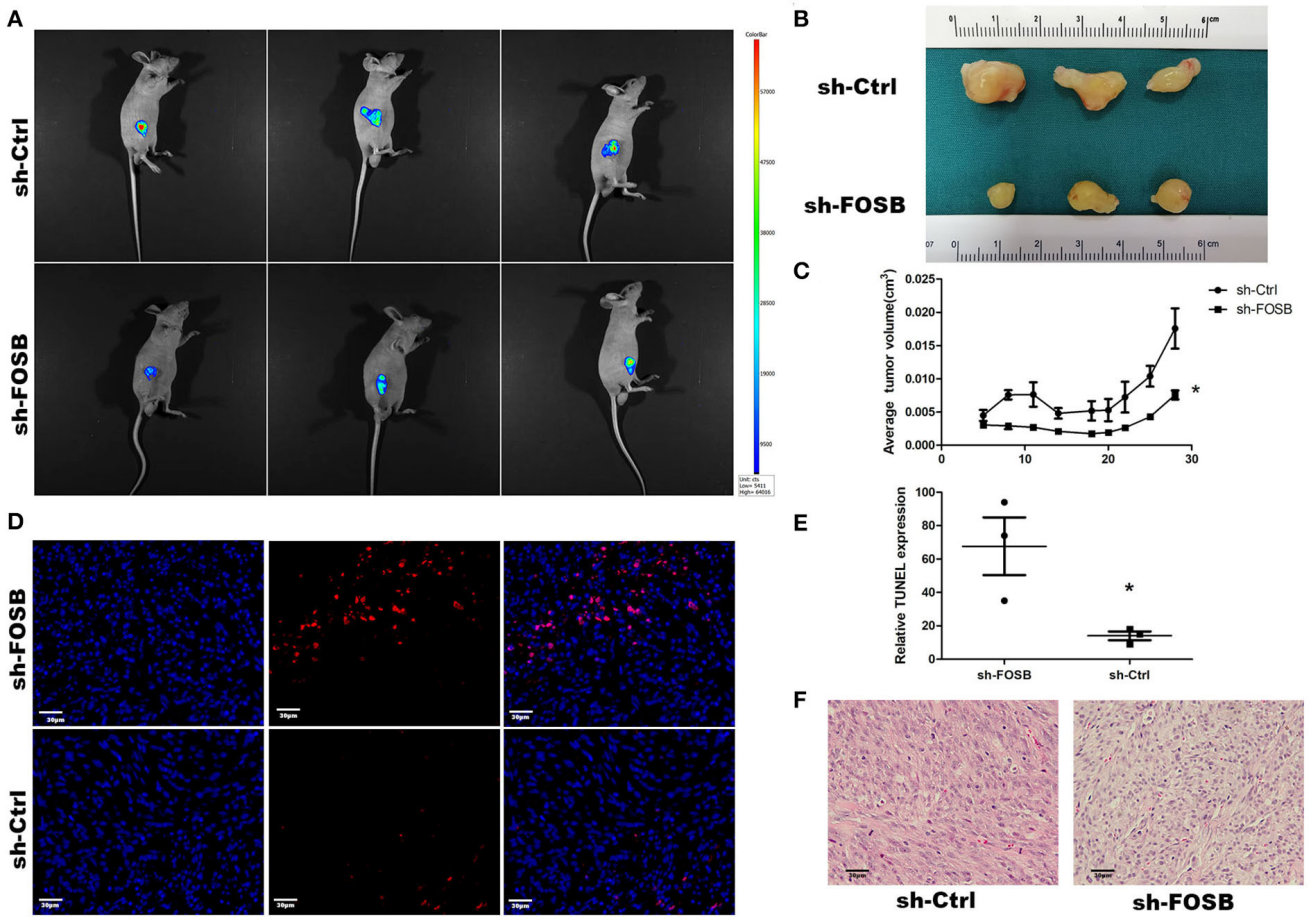
*In vivo* effect of FOSB expression on promoting the growth of mouse GBM cell xenografts. **(A)** Imaging of mice. The nude mice of the sh-FOSB group and sh-Ctrl group on the 28th day of subcutaneous implantation were photographed through the Small Animal *in-vivo* Image system. The picture shows the subcutaneous tumor with GFP fluorescence. **(B)** After 28 days of GBM cell implantation, the tumor xenografts were removed from the mice and compared. **(C)** Tumor volume. The subcutaneous tumor-bearing volume of mice in the control and experimental groups was measured on days 5, 8, 11, 14, 18, 20, 22, 25, and 28, respectively (*n* = 3, *P < 0.05 vs. sh-Ctrl group). **(D)** TUNEL staining. The resected tumor xenografts were subjected to TUNEL staining. **(E)** It was the counting result of positive cells in **(D)**, *P < 0.05 vs. sh-Ctrl group. **(F)** Hematoxylin-eosin staining was performed in the experimental group and the experimental group after subcutaneous dissection.

The growth of glioma cell lines transplanted to nude mice was monitored by subcutaneous tumor xenografts in nude mice. The down-regulation of FOSB could affect the growth of glioma cells transplanted to nude mice. At present, the *U87* cell line has a good tumorigenic effect in nude mice, so we chose the *U87* glioma cell line. The measured data showed that FOSB decreased significantly after subcutaneously compared with the control group (*n* = 3; Figures 4A,B), and the growth rate of the subcutaneous tumor was slower than that of the control group (^*^P < 0.05; Figure 4C). Meanwhile, TUNEL staining showed that apoptosis increased significantly after FOSB knockdown (^*^P < 0.05; Figures 4D,E). HE staining showed that the cell atypia of *U87* cells decreased after FOSB knockdown (Figure 4F).

## Discussion

Glioma accounts for ~80% of primary malignant tumors of the central nervous system. The high recurrence rate of glioma exacerbates the prognosis of glioma patients. Even low-grade glioma tends to develop to a higher level (Malta et al., 2018). At present, surgical resection combined with TMZ and radiotherapy could only slightly prolong the survival of GBM patients. Therefore, clarifying the internal mechanism underlying its highly malignant properties is key to developing efficacious therapeutic regimens (Bandey et al., 2015). FOSB (AP-1 transcription factor subunit) is a proto-oncogene in many tumor types. It has been reported that silencing of Fos can promote cell cycle arrest and enhance GBM sensitivity to radiation. There is a lot of evidence that FOSB has an important role in regulating cell proliferation, differentiation, cell cycle, and transformation. However, the biological function of FOSB in glioma remains unclear.

Compared with normal brain tissues, we found that FOSB was highly expressed in glioma tissues both at the protein and mRNA levels. In addition, the expression of FOSB in high-grade glioma was much higher than that in low-grade glioma. Interestingly, FOSB expression in *U118* glioma cells was lower than that in normal HA, but FOSB mRNA expression was significantly higher in *U118* than in HA. There are many processes between transcription and translation, and protein stability is a big factor. Transcriptional data are useful for identifying potential candidates for follow-up at the protein level. However, changes in gene expression may not be reflected at the protein level.

We also found that downregulation of FOSB could effectively inhibit cell proliferation, colony formation, and cell invasion. Caspase proteins such as caspase-3 are considered “common suspects” of cell death. Cleaved caspase-3 is often used to reflect apoptosis (Shen et al., 2016; Sun et al., 2017; Weng et al., 2019). AP1 factors could regulate the process of proliferation, apoptosis, and differentiation (Rorke et al., 2010; Barrett et al., 2017). We also reported that adjustment of FOSB expression could significantly affect the expression of cleaved caspase-3; that is to say, FOSB could regulate apoptosis. Through a series of phenotypic experiments, we found that after the silencing of FOSB, the proliferation and migration ability of GBM cell lines were weakened. Interestingly, TMZ resistance significantly decreased when FOSB expression was downregulated in *U87* and *U251* cell lines.

As a limitation, we have not explored the upstream regulators of FOSB in the present study. In the literature, ERK1/2 is an upstream protein of FOSB. After phosphorylation, ERK1/2 can activate the Fos transcription factor protein family (Dhandapani et al., 2007; Giordano et al., 2016; Gazon et al., 2017). Phosphorylation of ERK1/2 can promote tumor cell development in glioma (Jin et al., 2018). It is necessary to further explore whether there is any interaction between FOSB expression and ERK1/2 or other upstream proteins. These experiments will be conducted in our laboratory (Supplementary Image 1).

In conclusion, we found that FOSB was overexpressed in human glioma tissues and GBM cell lines compared with normal human brain tissues and astrocyte lines, respectively. Moreover, downregulating FOSB could significantly reduce the proliferation, migration, and TMZ resistance of glioma. These findings suggest that FOSB may be a potential target for glioma therapy.

## Data availability statement

The datasets presented in this study can be found in online repositories. The names of the repository/repositories and accession number(s) can be found in the article/Supplementary material.

## Ethics statement

The studies involving human participants were reviewed and approved by Laboratory Animal Welfare and Ethics Committee of Yijishan Hospital. The patients/participants provided their written informed consent to participate in this study. The animal study was reviewed and approved by Laboratory Animal Welfare and Ethics Committee of Wannan Medical College.

## Author contributions

MQ, L-aS, M-lZ, X-cJ, and L-rZ concept carried out research, designed, and drafted the manuscript. MQ, L-aS, W-hN, and M-xF participated in cell experiments. MQ and JZ participated in sample collection and IHC staining. MQ, Y-lH, FW, JZ, M-lZ, X-cJ, and L-rZ participated in data analysis. All authors read and approved the final manuscript.

## Funding

This work was supported by the National Natural Science Foundation of China (grant numbers 81771292, 81571162, and 81472366) and the Natural Science Foundation of Anhui Province (grant number 1804h08020234).

## Conflict of interest

The authors declare that the research was conducted in the absence of any commercial or financial relationships that could be construed as a potential conflict of interest.

## Publisher's note

All claims expressed in this article are solely those of the authors and do not necessarily represent those of their affiliated organizations, or those of the publisher, the editors and the reviewers. Any product that may be evaluated in this article, or claim that may be made by its manufacturer, is not guaranteed or endorsed by the publisher.
