# Supplementary figures and images for: Expression and potential role of FOSB in glioma
Source: Front Mol Neurosci. 2022 Oct 12;15:972615. doi: 10.3389/fnmol.2022.972615 (PMC9597691; doi:10.3389/fnmol.2022.972615)

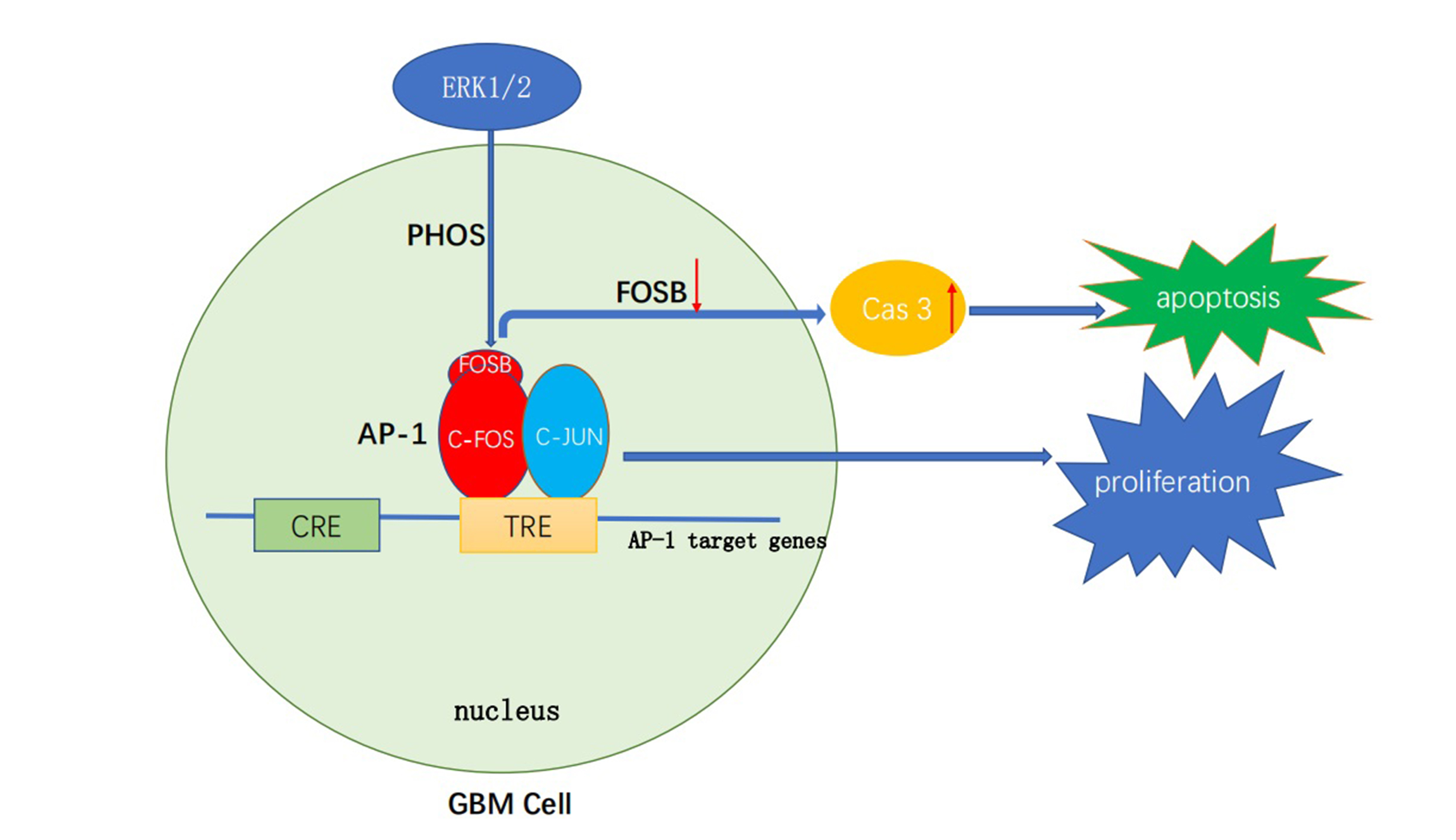

Supplement: Supplementary file 1 [file Image_1.JPEG]
